# Sensitivity analysis of cell fate trajectories from single-cell transcriptomics

**DOI:** 10.1093/bioinformatics/btag473

**Published:** 2026-08-21

**Authors:** Abdullah Al Noman, Palash Sashittal

**Affiliations:** Department of Computer Science, Virginia Tech, Blacksburg, VA 24060, United States; Department of Computer Science, Virginia Tech, Blacksburg, VA 24060, United States

## Abstract

**Summary:**

Cell differentiation is a dynamic process in which cells traverse through high-dimensional gene expression space under the influence of gene regulatory networks and environmental cues. Recent advances in single-cell RNA sequencing (scRNA-seq) have enabled us to measure high-resolution snapshots of this dynamic process. Several computational methods have been developed to reconstruct cellular flow maps from these snapshots, revealing the trajectories taken by cells in gene expression space. While existing methods provide increasingly detailed descriptions of cellular trajectories, the stability of these trajectories to perturbations is largely unexplored. As such, it remains unclear how robust inferred trajectories are to perturbations, which genes most strongly influence long-term fate outcomes, and where instability arises between competing fate commitments. While sensitivity and stability analysis tools from dynamical systems theory provide a principled way to study the stability of differentiation trajectories, existing approaches are not designed for the high-dimensionality and sparsity of scRNA-seq data. Here, we introduce FateSens, a sensitivity-based computational framework for analyzing gene regulatory dynamics using flow maps derived from scRNA-seq data. FateSens performs sensitivity analysis of differentiation trajectories derived from scRNA-seq data to identify regulatory genes and fate boundaries. To demonstrate its utility, we applied FateSens to study neutrophil-monocyte differentiation using scRNA-seq data of mouse hematopoiesis. While FateSens relies only on transcriptomic measurements, this dataset also contains lineage tracing barcodes that provide ground-truth fate relationships. Our results show that FateSens accurately recovers regulators consistent with known biology and identifies fate boundaries that are supported by lineage tracing data.

**Availability and implementation:**

We implement FateSens in Python 3, with an open-source implementation available at: https://github.com/sashittal-group/FateSens.

## 1 Introduction

Cell differentiation is a dynamic process in which cells transition between molecular states in response to intrinsic gene regulatory programs and extrinsic signals. These transitions underlie essential biological phenomena, including embryonic development, tissue homeostasis and regeneration, and cancer progression (Moris *et al*. 2016, Terryn *et al*. 2018). Since differentiation unfolds over time and involves coordinated changes across thousands of genes, it can be naturally described as a dynamical system (Teschendorff and Feinberg 2021). Inspired by the concept of the *Waddington landscape* (Waddington 2014), cell differentiation can be represented as trajectories through a high-dimensional space governed by an underlying flow determined by gene regulatory interactions. Modeling cell differentiation as a dynamical system holds great promise for advancing applications in tissue engineering and regenerative medicine by enabling design of strategies involving genetic and chemical perturbations to direct cell-fate outcomes (Macarthur *et al*. 2009, Kamimoto *et al*. 2023).

Single-cell RNA sequencing (scRNA-seq) has revolutionized our ability to study biological processes by providing high-resolution snapshots of the dynamics of gene expression of individual cells. Several computational methods have been developed employing various approaches to infer flow maps that approximate how cells move through gene expression space over time using scRNA-seq data. For multi-timepoint scRNA-seq data, optimal transport–based approaches such as Moscot (Klein *et al*. 2025), Waddington-OT (Schiebinger *et al*. 2019), CoSpar (Wang *et al*. 2022) connect cells across longitudinal measurements to estimate transition probabilities between states. In single-timepoint data, pseudotime and RNA velocity-based methods like Cellrank (Lange *et al*. 2022), Cytopath (Gupta *et al*. 2022), Vetra (Weng *et al*. 2021) leverage asynchronicity in differentiation across cells and splicing kinetics to infer local directionality of state transitions which can be integrated to reconstruct differentiation trajectories. More recently, generative and dynamical modeling approaches have been proposed to learn transition operators that predict future cellular states from current states (Yeo *et al*. 2021). While the flow maps inferred by these methods provide crucial insights about cellular dynamics, the stability of these trajectories remains an underexplored area. Analyzing the stability of differentiation trajectories will allow us to characterize how robust the inferred trajectories are to perturbations and which genes most strongly influence long-term fate outcomes.

A principled way to studying stability of dynamic trajectories is to perform perturbation analysis, i.e. examining how small changes in system variables alter long-term behavior. In the context of cell differentiation, perturbation analysis would provide causal insight into how gene expression changes influence developmental outcomes. Recent advances in high-throughput CRISPR-based screening technologies coupled with single-cell transcriptomics, have enabled large-scale interrogation of perturbations in gene expression on cellular state transitions (Bock *et al*. 2022). Despite these advances, experimental perturbation studies remain expensive, technically demanding, and inherently limited in scope. Specifically, only certain classes of perturbations, such as monogenic knockouts (Dixit *et al*. 2016), targeted transcriptional interference (Adamson *et al*. 2016) and activation (Jaitin *et al*. 2016) can be implemented reliably and combinatorial interventions scale poorly. These limitations highlight the need for computational frameworks that enable principled perturbation analysis that can complement experimental screens, prioritize candidate regulators, and provide mechanistic insight into the principles governing cell differentiation.

The mathematical foundations for such computational frameworks are well established. Stability and sensitivity analysis are foundational tools in dynamical systems theory and have been widely applied to physical and engineered systems, including aerospace control systems (Stevens *et al*. 2015), fluid dynamics (Schmid *et al*. 2002) and climate modelling (Dijkstra 2013). While several tools have been developed to study these complex dynamical systems, the unique features of cellular differentiation dynamics and scRNA-seq data make direct application of these classical tools challenging. In contrast to many engineered systems where governing equations are known, cellular dynamics must be inferred from noisy, high-dimensional observational data without an explicit parametric model. While data-driven system identification and stability analysis approaches have been developed before, they are not designed for the high-dimensionality and high rates of missing data (up to 75% dropout (Hu *et al*. 2020)) exhibited by scRNA-seq data. As such, there is a need for novel computational methods that would enable robust application of these principles from stability analysis of dynamical systems to single-cell transcriptomics.

We use principles from stability and sensitivity analysis of dynamical systems to mathematically describe fundamental biological concepts, enabling their systematic and quantitative characterization from single-cell data. We define differentiation regulatory genes as those whose perturbation most strongly alters long-term cell fate outcomes. Formally, these are genes to which the inferred dynamical system is most sensitive: small changes in their expression produce large shifts in a cell’s eventual fate. We further define fate boundaries as regions of gene expression space where developmental trajectories are maximally sensitive to perturbation and where small changes in state redirect cells toward distinct terminal fates. In dynamical systems terms, these boundaries correspond to high-sensitivity ridges that separate basins of attraction associated with different cell types.

In this work, we introduce FateSens, a novel sensitivity-based framework to analyze stability of differentiation trajectories inferred from scRNA-seq data. Applying FateSens to mouse hematopoiesis lineage tracing data (Weinreb *et al*. 2020), we identify fate boundaries within the neutrophil-monocyte trajectory and recapitulate established cell fate regulators across key branching events. By benchmarking our results against differentially expressed genes (DEGs) derived lineage-specific progenitors determined using lineage barcodes, we demonstrate that FateSens accurately identifies lineage-specific regulatory drivers. Furthermore, we verify that the fate boundaries predicted by our model segregate the progenitor cells in a way that aligns well with the empirical commitment-bias revealed by lineage tracing data.

## 2 Problem statements

Cell differentiation is a complex dynamical system in which cells change their state, characterized by expression of their genes. Let *g* denote the number of genes and let $x(t)\in R^{g}$ denote the state of a cell at time *t*. Following previous works (La Manno *et al*. 2018, Bergen *et al*. 2020), we model cell differentiation as a dynamical system dx/dt=v(x), where $v:R^{g}\to R^{g}$ is the velocity field specifying the instantaneous rate of change in gene expression as a function of the current state. The associated *flow map* $F_{t_{0}}^{t}:R^{g}\to R^{g}$ characterizes the Lagrangian transport of this dynamic system and defines how a cell at an initial gene expression $x_{0}$ evolves to its state at a later time *t*, i.e. $F_{t_{0}}^{t}(x_{0})=x(t)$ with the identity property $F_{t_{0}}^{t_{0}}(x_{0})=x_{0}$. When the inital time is clear from context, we write $F^{t}$ and denote the evolved state as x(t). The flow map encapsulates the cumulative integration of the velocity field from the initial to the final state.

In practice, the true flow map governing cellular differentiation is not directly observable. Single-cell RNA sequencing (scRNA-seq) is inherently destructive: each cell is measured once and cannot be followed longitudinally. As a result, we do not observe individual trajectories, but rather population-level snapshots of cells sampled at discrete time points. Suppose we sample $n_{0}$ and $n_{1}$ cells at two time points $t_{0}$ and $t_{1}$ (where $t_{1}>t_{0}$), respectively. Using scRNA-seq, we measure the gene expression matrices $X\in R^{n_{0}\times g}$ and $X^{\prime }\in R^{n_{1}\times g}$, where each row $x_{i}$ in *X* and $x_{j}^{\prime }$ in $X^{\prime }$ represent the gene expression of cell *i* from time point $t_{0}$ and cell *j* from time point $t_{1}$, respectively. Reconstructing the flow map therefore requires inferring how cells at an earlier time relate to cells observed later.

Beyond reconstructing the flow map itself, a central objective is to understand how perturbations at an initial time influence biologically meaningful outcomes at a later time. This question is formalized through the sensitivity of the flow map. The Jacobian of the flow map $F^{t}$ with respect to the initial condition $x_{0}$ is $J(x_{0})=\nabla F^{t}(x_{0})\in R^{g\times g}$, where each entry of $J(x_{0})$ is


$$J_{i,j}(x_{0})=\frac{\partial x_{i}(t)}{\partial x_{0,j}}.$$

This operator governs the evolution of infinitesimal perturbations. A small perturbation $\delta x_{0}$ in the initial state induces, to first order, change $\delta x(t)\approx J(x_{0})\delta x_{0}$ in the final state x(t).

While the Jacobian describes sensitivity of the entire gene expression state, in many applications we are interested in the sensitivity of a specific downstream quantity. Let $f:R^{g}\to R^{k}$ denote a function of interest evaluated at the terminal state, such as the expression level of marker genes, or the probability of a cell belonging to a particular fate. We are interested in the composition $x_{0}\mapsto f(F^{t}(x_{0}))$, and we seek to quantify how perturbations in the initial state affect this downstream outcome. The local sensitivity of this quantity with respect to the initial condition is given by the Jacobian $S_{f}(x)=\nabla (f\;{}^{\circ}\;F^{t})(x_{0})$. This quantity provides the sensitivity of the functional output at time *t* with respect to perturbations at time $t_{0}$. We formally pose the problem of estimating the function senstivity matrix as follows.

**Problem 2.1** (Function Sensitivity Estimation). *Suppose we are given gene expression matrices X and* $X^{\prime }$  *of* $n_{0}$  *and* $n_{1}$  *cells from time* $t_{0}$  *and* $t_{1}$*, respectively, generated by an unknown flow map* $F_{t_{0}}^{t_{1}}:R^{g}\to R^{g}$*. Let* $f:R^{g}\to R^{k}$  *be a function of interest defined on the terminal states. For a given initial state*  $x_{0}\in R^{g}$*, estimate the function sensitivity matrix* $S_{f}(x)=\nabla (f\;{}^{\circ}\;F^{t})(x_{0})$  *using X and* $X^{\prime }$.

Besides local sensitivity, an important biological concept is fate boundaries, i.e. regions in gene expression space where small perturbations in the progenitor state lead to large differences in terminal outcomes. Intuitively, these are boundaries in gene expression space separating basins of attraction corresponding to distinct cell fates. Cells initialized on opposite sides of such boundaries evolve toward different terminal states.

In dynamical systems theory, a closely related concept used to study fluid dynamical systems is finite-time Lyapunov exponent (FTLE) (Mowlavi *et al*. 2022). For a flow map $F_{t_{0}}^{t_{1}}$ with Jacobian $J(x_{0})=\nabla_{x_{0}}F_{t_{0}}^{t_{1}}(x_{0})$, the FTLE at $x_{0}$ over the interval $\left[t_{0},t_{1}\right]$ is defined as


$$\Lambda_{t_{0}}^{t_{1}}(x_{0})=\frac{1}{\left|t_{1}-t_{0}\right|}\ln\left(\max_{\delta x_{0}\neq 0}\frac{\left\|J(x_{0})\delta x_{0}\right\|}{\left\|\delta x_{0}\right\|}\right).$$

The maximization term equals the spectral norm of $J(x_{0})$, i.e. the largest singular value of $J(x_{0})$. Let $J=U\Sigma V^{⊤}$ be the singular value decomposition of *J*, where Σ is a diagonal matrix with singular values $\sigma_{1}\geq \sigma_{2}\geq \cdots \geq \sigma_{g}\geq 0$. The FTLE simplifies to


$$\Lambda_{t_{0}}^{t_{1}}(x_{0})=\frac{1}{\left|t_{1}-t_{0}\right|}\ln\sigma_{1}(x_{0}).$$

Regions with high FTLE values correspond to strong local stretching of trajectories: infinitesimally close initial states diverge maximally along the dominant singular direction. In fluid dynamics, ridges of the FTLE field define Lagrangian coherent structures, which act as transport barriers separating dynamically distinct regions (Mowlavi *et al*. 2022).

In the context of cell differentiation, we are interested in regions that separate fate decisions defined by a specific function f(x) of the terminal state x. Analogous to the classical FTLE, we define the functional finite-time amplification rate


$$\Lambda_{f}(x_{0})=\frac{1}{\left|t_{1}-t_{0}\right|}\ln\left(\left|S_{f}(x_{0})\right|\right).$$

This quantity measures the maximal amplification of infinitesimal perturbations in the initial gene expression state as observed through the functional *f*.

We define fate boundaries as ridges of the functional FTLE field $\Lambda_{f}(x)$. Intuitively, these are regions where infinitesimal perturbations produce the largest changes in the outcome of interest. Following previous work (Zhang *et al*. 2022), a ridge of $\Lambda_{f}$ is defined as a set $R\subset R^{g}$ of points such that $V_{E}{\left(x\right)}^{⊤}\nabla \Lambda_{f}(x)=0$, where $V_{E}$ are the eigenvectors of $\nabla^{2}\Lambda_{f}(x)$ having eigenvalues less than zero. Zero-dimensional ridges correspond to isolated unstable points, while higher-dimensional ridges form curves or hypersurfaces that partition state space into regions of distinct dynamical behavior. We formally pose the problem of identifying fate boundaries from gene expression data as follows.

**Problem 2.2** (Fate Boundary Identification). *Let X and* $X^{\prime }$  *be gene expression matrices of* $n_{0}$  *and* $n_{1}$  *cells from time* $t_{0}$  *and* $t_{1}$*, respective, generated by an unknown flow map* $F_{t_{0}}^{t_{1}}$*. Given a function* $f:R^{g}\to R^{k}$*, identify the fate boundaries characterized by ridge set of* $\Lambda_{f}$  *associated with function f.*

## 3 FateSens: sensitivity analysis of differentiation trajectories

**Figure 1:**
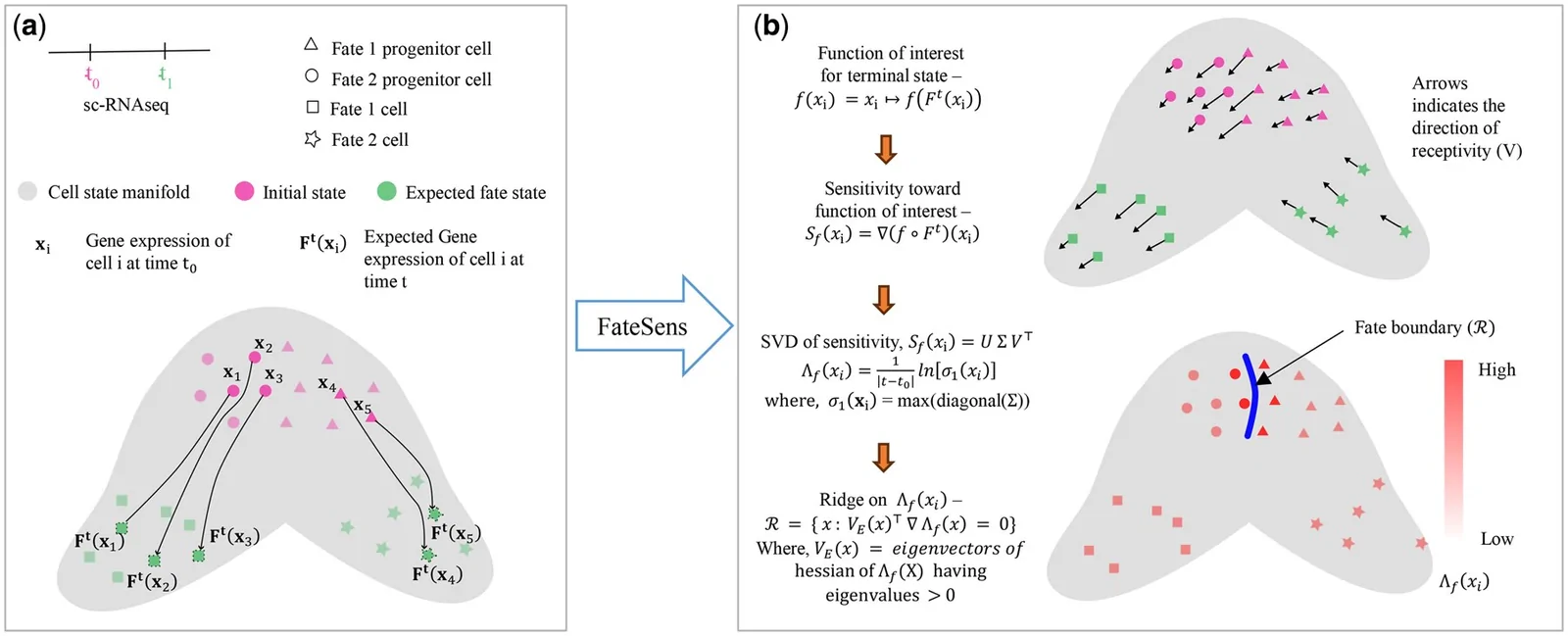
Schematic overview of the FateSens framework. (a) FateSens takes scRNA-seq data sampled at two distinct timepoints $t_{0}$ and $t_{1}$ and the inferred flow map $F^{t}(x_{i})$ for the sampled cells as input. (b) FateSens estimates the function sensitivity matrix $S_{f}(x_{i})$ of function *f* (evaluated at terminal fate) with respect to the initial cell state. Fate boundary R is calculated by identifying a ridge on the functional finite-time amplification rate $\Lambda_{f}$ (shown by the solid blue line on the manifold) of function *f*.

We introduce FateSens (Fig. 1), a framework for sensitivity analysis of differentiation trajectories. FateSens has three modules: (1) function sensitivity estimation, (2) fate probability sensitivity estimation, and (3) fate boundary identification of specific cell fate decisions.

### 3.1 Function sensitivity estimation

In this section, we describe our algorithm to estimate the sensitivity of a given function *f* of terminal state to perturbation to initial state from scRNA-seq data. A central step to solve Function Sensitivity Estimation problem (Prob. 2.1) is inference of the underlying flow map from scRNA-seq data across multiple time points.

Several methods have been developed that approximate the underlying flow by estimating a coupling matrix $T\in {\left[0,1\right]}^{n_{0}\times n_{1}}$ that probabilistically links cells across time points(Schiebinger *et al*. 2019, Wang *et al*. 2022, Klein *et al*. 2025). Specifically, each entry $T_{ij}$ represents the probability that a cell *i* with state $x_{i}$ transitions to a state resembling $x_{j}^{\prime }$ of cell *j* at time $t_{1}$, thereby providing a discrete approximation to the action of the continuous flow map over the interval $t_{1}-t_{0}$. By construction, *T* is row stochastic, i.e. $\sum_{j=1}^{n_{1}}T_{i,j}=1$. The coupling matrix *T* provides a discrete approximation to the action of the flow map on cells sampled at time $t_{0}$ over the interval $\left[t_{0},t_{1}\right]$. Specifically, each row of *T* as a probability distribution over possible future states. For a cell with initial state $x_{i}$ at time $t_{0}$, the expected state at time $t_{1}$ is given by $E[F^{t}(x_{i})]=\sum_{j=1}^{n_{1}}T_{ij}x_{j}^{\prime }$.

Our goal is to quantify how small perturbations in a cell’s state x influence some function *f* of the future state, i.e. the Jacobian of $\left(f{}^{\circ}F^{t})(x\right)$. A major challenge in accurately estimating this is that scRNA-seq measurements are sparse with respect to the high dimensionality of gene expression space and error-prone. We overcome this by adopting a local linear regression framework to estimate the Jacobian. Specifically, for any point x, we assume that the Jacobian is constant within a neighborhood around x, so that f(x+δx)≈f(x)+Δf(x)δx, where ∇f(x) is the Jacobian of *f* at x. The problem reduces to estimating the best linear operator that explains the observed local deformations.

Let $N_{\delta }(x)$ denote the neighborhood around point x, i.e. the set of cells that are within a distance of δ from x. For any two cells *i* and *j* in $N_{\delta }(x)$, we define the displacement vectors $\Delta x_{i,j}=x_{i}-x_{j}$ and $\Delta f_{i,j}=f(x_{i})-f(x_{j})$. If f(x) is locally linear, then $\Delta f_{i,j}=\nabla f(x)\Delta x_{i,j}$, where ∇f(x) is the Jacobian of *f* at x. We estimate ∇f(x) by solving the Tikhonov-regularized least-squares problem (Mowlavi *et al*. 2022),


$$\hat{\Delta f}(x)=\underset{A}{\arg\min}\left(\sum_{j\neq k\in N_{i}}\|A\Delta x_{jk}-\Delta f_{jk}{\|}_{2}^{2}+\beta \|A{\|}_{F}^{2}\right).$$

Here, β>0 is a regularization parameter. Let $D_{X}$ and $D_{f}$ be matrices whose columns are pairwise differences $\Delta x_{ij}$ and $\Delta f_{i,j}$ for all unique pairs of cells in $N_{\delta }(x)$. The minimizer admits the closed-form solution $\hat{\nabla f}(x)=D_{f}D_{X}^{⊤}{\left(D_{X}D_{X}^{⊤}+\beta I\right)}^{-1}$. This formulation leverages all pairwise displacements within the neighborhood, thereby reducing sensitivity to noise in any individual cell and yielding a robust local estimate of how perturbations in gene expression propagate forward in time.

### 3.2 Fate probability sensitivity estimation

In several applications, it is beneficial to focus on cell type commitment rather than continuous state evolution. Suppose the cells at time $t_{1}$ are classified into *k* cell types, and let $P^{\prime }\in {\left[0,1\right]}^{n_{1}\times k}$ denote the matrix of cell-type membership probabilities, where $p_{j,\ell }^{\prime }$ is the probability that cell *j* belongs to cell type ℓ. The coupling matrix *T* induces a pushforward of these probabilities to time $t_{0}$, given by $P=TP^{\prime }$, where each entry $p_{i,\ell }$ of *P* represents the probability that a cell starting in state $x_{i}$ at time $t_{0}$ attains cell type ℓ at time $t_{1}$. In this formulation, the flow map acts as a linear operator transporting probability mass between cell states and, consequently, between cell fates. We will denote the cell fate probability of cell with state x at time $t_{0}$ as P(x).

The estimation of ∇P(x) proceeds analogously to the previous section, but must respect the simplex constraint $\sum_{\ell =1}^{k}P_{\ell }(x)=1$. Differentiating this identity implies $1^{⊤}\nabla P(x)=0$, so the rows of the Jacobian must sum to zero.

Let $A\in R^{k\times G}$ denote the Jacobian of *P*. Using pairwise differences $\Delta P_{ij}=P(x_{i})-P(x_{j})$, and assembling displacement matrices $D_{X}$ and $D_{P}$, we solve


$$\underset{A}{\min}\left(\|AD_{X}-D_{P}{\|}_{F}^{2}+\beta \|A{\|}_{F}^{2}\right)\;\text{subject}\;\text{to}\;1^{⊤}A=0.$$

This is the well-studied quadratic least-squares problem with linear equality constraints (Nocedal and Wright 2006). Introducing Lagrange multipliers $\lambda \in R^{G}$, yields the linear system


$$\begin{array}{c} \left[\begin{array}{cc} D_{X}D_{X}^{⊤}+\beta I & 1\\ 1^{⊤} & 0 \end{array}\right]\left[\begin{array}{c} A^{⊤}\\ \lambda^{⊤} \end{array}\right]=\left[\begin{array}{c} D_{X}D_{P}^{⊤}\\ 0 \end{array}\right], \end{array}$$

Whose solution provides the constrained estimator $\hat{\nabla P}(x)$ (algorithmic details in Supplemental Section 8, available as supplementary data at *Bioinformatics* online). By enforcing probability conservation at the level of the differential operator, this approach ensures that first-order perturbations preserve total mass, while quantifying how small expression changes redistribute probability among competing fates.

### 3.3 Fate boundary identification

In this section, we describe our algorithm to solve the Fate Boundary Identification problem (Prob. 2.2). For a given function *f*, we use the least square regression-based approach described in the previous section to compute the function sensitivity matrix $S_{f}(x)$ for all $n_{0}$ cells at time $t_{0}$. Singular value decomposition of this matrix at each cell is used to compute the finite-time amplification rate $\Lambda_{f}$. Estimating fate boundaries reduces to identifying ridges in the field $\Lambda_{f}(x)$ given its values sampled at $n_{0}$ points in gene expression space corresponding to cells at time $t_{0}$. We use the Subspace Constrained Mean Shift (SCMS) algorithm (Ozertem and Erdogmus 2011) for the ridge identification. We provide details of the algorithm and its application in Supplemental Section 3, available as supplementary data at *Bioinformatics* online.

## 4 Results

### 4.1 Longitudinal single-cell lineage tracing data of mouse hematopoiesis

We apply FateSens to analyze sensitivity of differentiation trajectories during mouse hematopoiesis using a longitudinal single-cell lineage tracing dataset generated by (Weinreb *et al*. 2020). This dataset couples single-cell RNA sequencing with heritable barcode information, enabling reconstruction of clonal relationships alongside transcriptional states across time. This data comprises 1 30 887 single cells profiled at three discrete time points (days 2, 4, and 6), with clonal barcodes recovered for 49,302 cells. In our study, we use flow maps inferred on both temporal intervals $\left[t_{0}=2,t_{1}=4\right]$ and $\left[t_{0}=4,t_{1}=6\right]$ using WaddingtonOT (Schiebinger *et al*. 2019), each corresponding to an elapsed time of Δt=2 days (details in the Supplemental Section 4, available as supplementary data at *Bioinformatics* online). Considering both intervals enables us to evaluate FateSens across distinct stages of hematopoietic differentiation while maintaining a consistent time horizon. The data consists of 23 770 undifferentiated hematopoietic stem cells, and differentiated cells from 9 cell types: 8555 neutrophils, 8165 monocytes, 1035 megakaryoctyes, 365 erythrocytes, 1414 mast cells, 5514 basophils, 168 eosinophils and 113 dendritic cells. We focus our analysis on neutrophil-monocyte, which comprise of the two most abundant cell types in the dataset. This dataset provides a rigorous benchmark for FateSens. The simultaneous and longitudinal measurements of gene expression and clonal relationships enables linking cell state to eventual fate, which is precisely the setting required to evaluate sensitivity of differentiation outcomes to perturbations in initial state inferred by FateSens.

### 4.2 Sensitivity analysis of neutrophil-monocyte trajectories reveal differentiation regulatory genes

We use FateSens to identify regulators of neutrophil–monocyte commitment using sensitivity of a lineage balance function $f_{\text{mono}-\text{neu}}$ that increases with expression of monocyte marker genes and decreases with expression of neutrophil marker genes,


$$f_{\text{mono}-\text{neu}}(x)=f_{\text{mono}}(x)-f_{\text{neu}}(x)=\sum_{j\in M_{\text{mono}}}x_{j}-\sum_{j\in M_{\text{neu}}}x_{j},$$

Where $f_{\text{mono}}(x)$ and $f_{\text{neu}}(x)$ are the total expression of monocyte marker genes $M_{\text{mono}}$ and neutrophil marker genes $M_{\text{neu}}$, respectively (Details in Supplemental Section 5, available as supplementary data at *Bioinformatics* online). Genes with high positive sensitivity are predicted to promote monocytic differentiation while simultaneously suppressing neutrophilic differentiation. In contrast, genes with high negative sensitivity are predicted to promote neutrophilic differentiation and suppress monocytic commitment. We define cell-type–specific differentiation regulatory genes as those with the largest magnitude sensitivities in either direction.

We examine if these sensitivity-defined regulators are consistent with independent evidence of lineage bias derived from the clonal information in the data. The clonal barcodes in this dataset enable quantification of progenitor commitment bias by providing an estimate for the fraction of descendant cells of the progenitor adopting neutrophil versus monocyte fates. Progenitors whose descendants are predominantly neutrophils are labeled neutrophil-biased, whereas those whose descendants are predominantly monocytes are labeled monocyte-biased. Ground-truth regulatory genes are identified performing differential gene expression analysis on neutrophil-biased and monocyte-biased progenitors. We identified 81 genes significantly overexpressed in neutrophil-biased progenitors and 32 for monocyte-biased progenitors (*P*value threshold 0.05 and logfold ratio 0.5, details in Supplemental Section 2, available as supplementary data at *Bioinformatics* online).

Differentiation regulatory genes identified by FateSens substantially overlap with genes differentially expressed between neutrophil-biased and monocyte-biased progenitors. Specifically, genes predicted to promote neutrophil differentiation (negative sensitivity) are preferentially overexpressed in neutrophil-biased progenitors (normalized area under the intersection-over-union (IOU) curve = 0.18), whereas genes predicted to promote monocyte differentiation (positive sensitivity) are enriched among monocyte-biased progenitors (area under IOU = 0.36). In contrast, driver genes identified by WaddingtonOT exhibit weaker agreement with genes differentially expressed between neutrophil- and monocyte-biased progenitors (area under IOU = 0.02 for neutrophils and 0.06 for monocytes). This demonstrates that FateSens sensitivity scores capture biologically meaningful drivers of lineage commitment.

**Figure 2:**
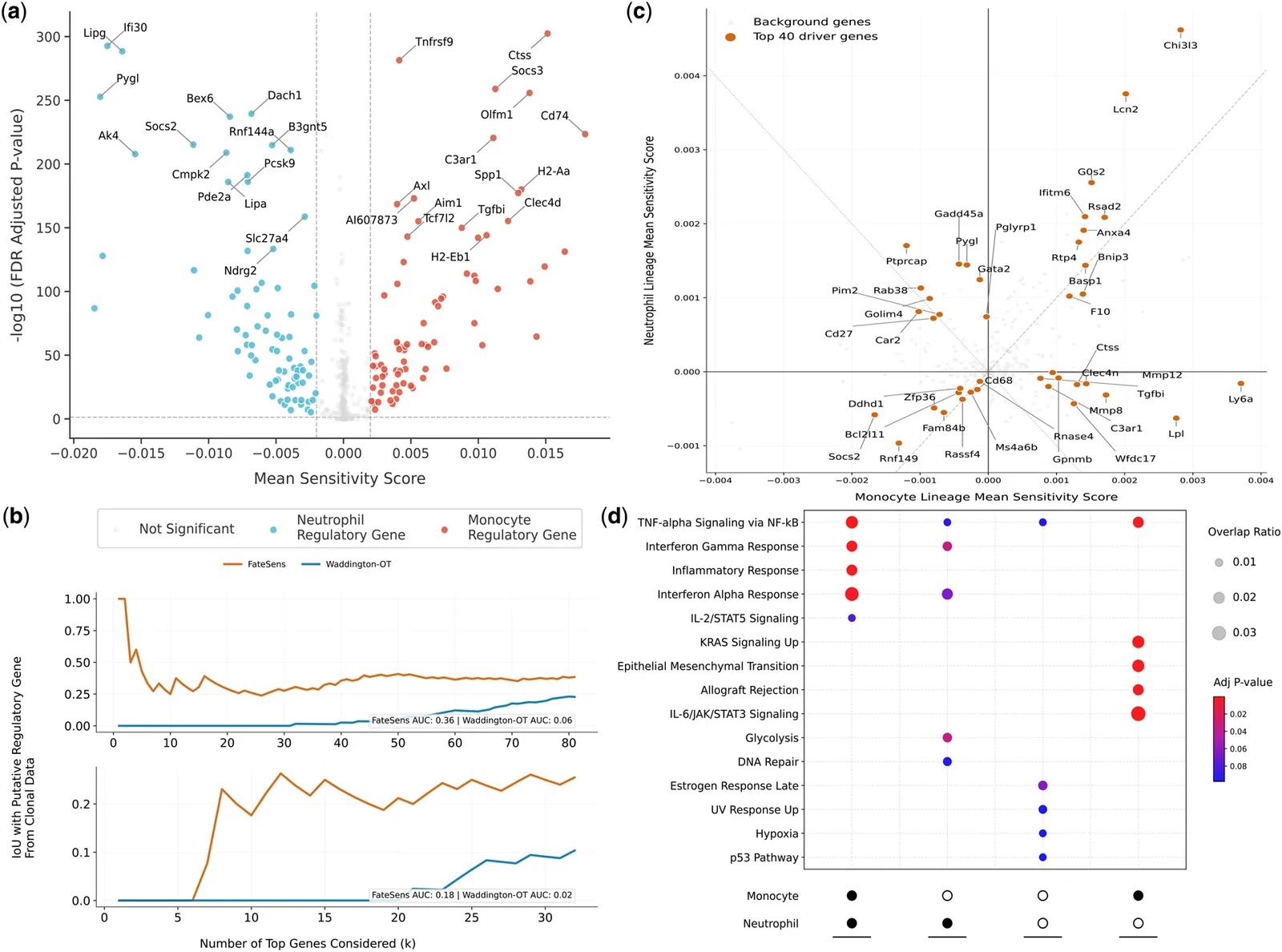
In-Silico sensitivity analysis of Neutrophil-Monocyte differentiation trajectories. (a) Statistical significance of regulatory genes across the neutrophil-monocyte fate transition. (b) Intersection over Union (IoU) of regulatory genes predicted by FateSens and Waddington-OT with putative regulatory genes derived from clonal data across an increasing number *k* of top regulatory genes considered for both monocyte and neutrophil differentiation. (c) Sensitivity analysis of neutrophil-monocyte differentiation mapped across four regulatory quadrants showing shared common regulators (+,+), shared common repressors (−,−), and mutually exclusive fate-specific drivers (+,− and −,+). (d) Gene set enrichment by expression profile on the four quadrants in (c).

Fate Sens provides a framework to dissect the regulatory roles of individual genes in neutrophil and monocyte differentiation (Fig. 2c and d). We classify genes into four regulatory roles based on the signs of their neutrophil and monocyte sensitivity scores: promoters of both fates (++), neutrophil-specific promoters that suppress monocyte differentiation (+−), monocyte-specific promoters that suppress neutrophil differentiation (−+), and shared repressors of both trajectories (−−) Gene set enrichment analysis (details in Supplemental Section 6, available as supplementary data at *Bioinformatics* online) across these four groups reveals that each regulatory class corresponds to distinct biological programs. Monocyte-specific regulators (+−) are enriched for pathways such as allograft rejection, consistent with programs associated with monocytic activation (Oberbarnscheidt *et al*. 2014), while, In contrast, neutrophil-specific regulators (−+) are enriched for inflammatory pathways, such as glycolysis for basal energy production, reflecting hallmark neutrophil effector functions (Morrison *et al*. 2023). Notably, genes that repress both trajectories (−−) are enriched for stress-associated programs, including hypoxia and the p53 pathway, which are known to induce cell cycle arrest (Hammond *et al*. 2002) and apoptosis (Leszczynska *et al*. 2015). These results demonstrate that FateSens not only identifies key lineage regulators but also systematically maps the functional divergence and shared biological dependencies underlying monocyte and neutrophil differentiation.

### 4.3 Fate boundaries inferred by FateSens separate progenitors with distinct fate bias

We use FateSens to identify the fate boundary for neutrophil-monocyte differentiation by extracting the ridge of the finite-time amplification rate $\Lambda_{P}$ of fate probability *P* (Sec. 3.2) of transitioning to neutrophils (probability of transitioning to monocytes is given by 1−P). We compared this to Waddington-OT; since it lacks explicit boundary definitions, we approximated its boundary using cells with nearly equiprobable fate commitments (0.49<P<0.51).

**Figure 3:**
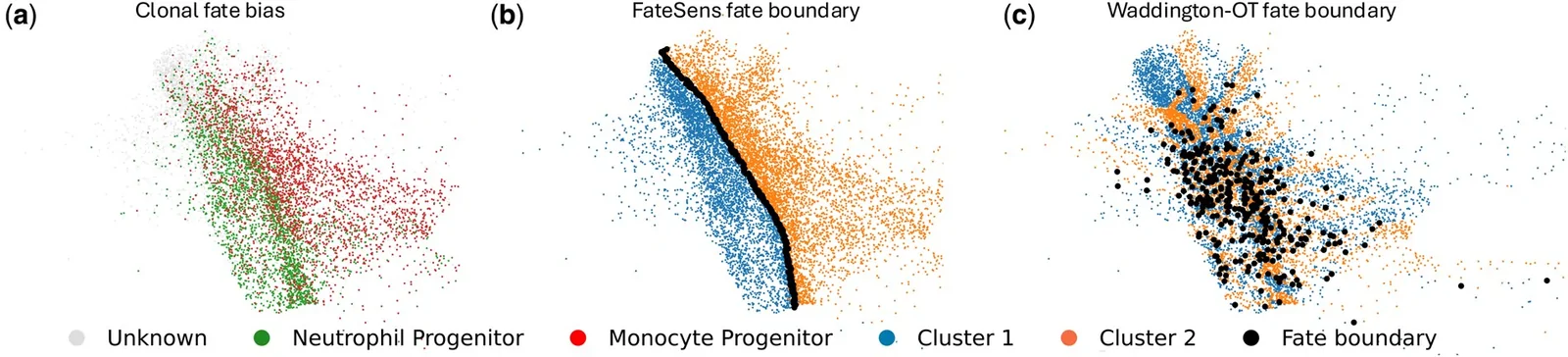
Fate Sens more accurate captures the bifurcation of neutrophil-monocyte differentiation compared to Waddington-OT. (a) Clonal fate bias determined for neutrophil and monocyte progenitors using lineage barcodes. Partition on progenitor cells induced by fate boundaries inferred using (b) FateSens and (c) Waddington-OT. All plots show two-dimensional projection of gene expression of 9737 progenitor cells generated using SPRING (Weinreb *et al*. 2018).

The FateSens fate boundary more accurate separates progenitor cells with distinct fate compared to the boundary obtained from Waddington-OT (Fig. 3). To evaluate how well the inferred fate boundaries partition the progenitor cells according to their commitment bias, we assign cells to boundary-derived clusters based on their geometric position relative to the inferred boundary using a geometric orientation test (details in Supplemental Section 7, available as supplementary data at *Bioinformatics* online). We then compare these partitions with lineage commitment inferred from independent lineage barcode measurements in the dataset. FateSens boundary-derived clusters (cluster 1 and cluster 2) agree more closely with the fate bias derived from lineage barcode information in the data compared to Waddington-OT (Table 1 and Supplemental Table 2, available as supplementary data at *Bioinformatics* online). Specifically, FateSens robustly segregates the lineages, placing 2281 monocyte progenitors into Cluster 2 and 1570 neutrophil progenitors into Cluster 1, while Waddington-OT-derived boundary splits the populations almost evenly across clusters, acting nearly as a random classifier. FateSens achieves an overall F1-score of 0.69, significantly outperforming Waddington-OT’s 0.53 (F1-score 0.73 vs 0.53 for monocytes and 0.65 vs 0.53 for neutrophils; Table 1). This demonstrates FateSens’s superior ability to a boundary that accurately capture the bifurcation of neutrophil-monocyte fate commitments.

**Table 1:** Performance of FateSens and Waddington-OT in separating cells based on fate bias.

Method	Cell population	Precision	Recall	F1-Score	Overall F1-Score
FateSens	Monocyte Progenitor	0.64	0.84	0.73	0.69
	Neutrophil Progenitor	0.78	0.55	0.65	
Waddington-OT	Monocyte Progenitor	0.51	0.55	0.53	0.53
	Neutrophil Progenitor	0.54	0.52	0.53	

## 5 Discussion

The FateSens framework introduces stability and sensitivity analysis techniques, traditionally utilized in dynamical systems and fluid dynamics, to the study of single-cell gene regulatory dynamics. We demonstrate on longitudinal single-cell RNA-sequencing data of in-vitro mouse hematopoiesis that FateSens that identify regulatory roles of genes in cell-type-specific differentiation and fate boundaries separating cells with different fate commitments.

There are several avenues for future research. First, although FateSens can quantify the sensitivity of any function of the terminal state to perturbations in individual genes, it does not explicitly model the underlying gene regulatory network. As a result, FateSens does not distinguish between direct regulatory interactions and indirect effects mediated through intermediate genes. An important direction for future work is to leverage these sensitivity estimates to reconstruct gene regulatory networks. Second, FateSens relies on existing methods for inference of the flow maps to model cellular dynamics, and inaccuracies in these estimates may propagate to the computed sensitivities. Inference of the flow map with sensitivity that represents an underlying regulatory structure could improve robustness and biological interpretability. Finally, extending this analysis to single-cell multi-omics data represents a particularly promising direction. Integrative multiomic sensitivity analysis would enable quantification of the sensitivity of one molecular layer to perturbations in another, for example, the sensitivity of gene expression to perturbations in chromatin accessibility. Cross-modal sensitivity analysis would provide a more comprehensive view of how regulatory programs are coordinated across molecular layers during cell fate decisions, and could help uncover the mechanistic basis of lineage specification at unprecedented resolution.

## Supplementary Material

btag473_Supplementary_Data

## Data Availability

The code, processed data, and analysis generated in this work are available in github at https://github.com/sashittal-group/FateSens and in zenodo at https://doi.org/10.5281/zenodo.21303926.
