# Supplementary material for: Sensitivity analysis of cell fate trajectories from single-cell transcriptomics
Source: Bioinformatics. 2026 Aug 21;42(Suppl 2):btag473. doi: 10.1093/bioinformatics/btag473 (PMC13501315; doi:10.1093/bioinformatics/btag473)
Supplement: Supplementary file 1 [file btag473_supplementary_data.pdf]

Supplementary Information for: Sensitivity Analysis of Cell Fate Trajectories from Single-Cell Transcriptomics

Abdullah Al Noman[1] and Palash Sashittal[1,†]

[1]Department of Computer Science, Virginia Tech, VA 24060

†Corresponding authors: Palash Sashittal (sashittal@vt.edu)

(1) Predicted clusters from fate boundary

Method	Ground Truth	Cluster 1	Cluster 2
**FateSens**	Monocyte Prog.	434	**2281**
	Neutrophil Prog.	**1570**	1272
**Waddington-OT**	Monocyte Prog.	1457	1213
	Neutrophil Prog.	1385	1444

Table 2 Confusion Matrices for Lineage Classification

(2) Quantification of clonal fate bias

The clonal fate bias of undifferentiated progenitor cells was determined by evaluating the differentiation outcomes of their respective clonal sisters. For a trajectory bifurcating into two distinct lineages ($Fate_1$ versus $Fate_2$), we calculated a raw bias score ($Score_{raw}$) based on the difference in sister cell fractions:

$$Score_{raw} = \frac{N_{Fate1} - N_{Fate2}}{N_{Total}} \tag{1}$$

where N_{Fate1} and N_{Fate2} represent the count of clonal sisters committed to each respective lineage. Cells lacking a clonal barcode or recovered sisters were assigned a baseline score of zero. Progenitors with raw scores above the median were classified as $Fate_1$ progenitors, while those below the median were designated as $Fate_2$ progenitors. The raw scores were smoothed over the transcriptomic manifold using K-nearest neighbor (K-NN) smoothing as suggested(Weinreb et al., 2020) for punitive regulator gene detection.

Identification of putative regulatory drivers

To characterize the molecular mechanisms underlying lineage specification, we identified the differentially expressed genes (DEGs) within the identified clonal progenitor populations. We assumed that the DEGs or highly correlated genes of these clonal progenitors represent a reasonable approximation of the true regulatory and driver genes for a given fate.

To systematically detect these regulatory genes, we subsetted the transcriptomic dataset to isolate undifferentiated cells that are $Fate_1$ and $Fate_2$ progenitors. Differential expression analysis was performed on this targeted subpopulation comparing the two type of fate progenitors using a Wilcoxon rank-sum test, implemented via the Scanpy library. Significant DEGs were subsequently extracted by applying a threshold requiring a Benjamini-Hochberg adjusted p-value < 0.05 and an absolute $\log_2$ fold change ≥ 0.5.

(3) Identification of Fate Boundaries

To explicitly identify the fate boundary of the differentiation landscape, we analyze the topological ridges of the finite-time amplification rate $\Lambda_f(x)$.

To extract these manifolds from the noisy, discrete transcriptome, we employ the Subspace Constrained Mean Shift (SCMS) algorithm(Zhang et al., 2022). While originally designed to detect principal curves (1D filaments) of probability densities, we adapt the method to locate $(G-1)$-dimensional ridges of the finite-time amplification rate field $\Lambda_f : \mathbb{R}^G \to \mathbb{R}$.

Let $\mathbf{H}(\mathbf{x}) = \nabla\nabla\Lambda_f(x)$ denote the Hessian of the amplification rate field. We consider its eigendecomposition with eigenvalues sorted $\lambda_1 \geq \cdots \geq \lambda_G$ and corresponding orthonormal eigenvectors $\mathbf{v}_1, \ldots, \mathbf{v}_G$. A fate boundary is characterized by a local maximum in the direction of greatest curvature. We define the projection subspace $\mathbf{V}_E(\mathbf{x}) = \mathbf{v}_G(\mathbf{x})$, corresponding to the eigenvector associated with λ_G.

The theoretical ridge set $\mathcal{R}$ is defined as the locus of points where the gradient vanishes in the direction of principal curvature:

$$\mathcal{R} = \{\mathbf{x} \in \mathbb{R}^G : \mathbf{V}_E(\mathbf{x})^\top \nabla\Lambda_f(x) = 0, \quad \lambda_G(\mathbf{x}) < 0\}. \tag{2}$$

In practice, we estimate $\Lambda_f(x)$ and its derivatives utilizing a Gaussian kernel smoother over the discrete cell population. The SCMS algorithm projects an arbitrary query point $\mathbf{x}^{(0)}$ onto the ridge via the iterative update rule:

$$\mathbf{x}^{(k+1)} \leftarrow \mathbf{x}^{(k)} + \eta \cdot \mathbf{V}_E(\mathbf{x}^{(k)})\mathbf{V}_E(\mathbf{x}^{(k)})^\top \nabla\hat{\Lambda_f}(\mathbf{x}^{(k)}). \tag{3}$$

This update climbs the gradient of the sensitivity field strictly along the local normal vector of the ridge, effectively converging to the separatrix that delineates distinct lineage domains.

(4) Constructing flow map

Preprocesssing mouse hematopoiesis LARRY data

The raw single-cell gene expression counts were preprocessed using the `Scanpy` to ensure comparability across cells and stabilize feature variance. First, we performed library-size normalization to a target sum of 10^4 counts per cell:

$$\tilde{x}_{ij} = \frac{x_{ij}}{\sum_{j=1}^{g} x_{ij}} \times 10^4 \tag{4}$$

where x_{ij} represents the raw count of gene j in cell i. To handle the high dynamic range and heteroscedasticity of the data, the normalized counts were log-transformed using the function $f(x) = \ln(1+x)$.

To reduce the dimensionality of the feature space and focus the sensitivity analysis on biologically informative signals, we identified the top 500 highly variable genes (HVGs) using the cell-conditioned dispersion method. The dataset was subsequently filtered to retain only these HVGs, resulting in a processed expression matrix $\mathbf{X} \in \mathbb{R}^{n\times 500}$ used for all subsequent trajectory and stability calculations.

Constructing flow map from coupling matrix

To capture the developmental trajectory across the observed time points, we first estimated the optimal transport couplings between sequential stages (Day 2 to Day 4, and Day 4 to Day 6) using Waddington-OT (Schiebinger et al., 2019). The model was parameterized with an entropic regularization parameter $\epsilon = 0.05$ and unbalanced transport penalties $\lambda_1 = 1$ and $\lambda_2 = 50$, iterating the growth estimates three times.

Let $\gamma_{2\to 4}$ and $\gamma_{4\to 6}$ denote the resulting local transport maps. To construct a unified transition model, we mapped the local cell indices to their global coordinates and assembled these local couplings into a comprehensive global sparse matrix $\mathbf{T}$. By design, this enforces strict chronological progression, resulting in a specific block-diagonal structure where non-sequential transitions (e.g., Day 2 directly to Day 6) are strictly zero (Table 3).

Source \Target	Day 4 Cells	Day 6 Cells
**Day 2 Cells**	Non-zero ($\gamma_{2\to 4}$)	0
**Day 4 Cells**	0	Non-zero ($\gamma_{4\to 6}$)

Table 3 Block structure of the global sparse transition matrix $\mathbf{T}$, illustrating that non-zero transition probabilities only exist for sequential, forward-time intervals between the source and target cells.

We define the continuous developmental flow map by row-normalizing this global matrix $\mathbf{T}$, such that it represents valid transition probabilities across time ($\sum_j T_{i,j} = 1$). For a cell with an initial state $\mathbf{x}_i$ at time t_0, its expected flow map state at the subsequent time point t_1 is computed as the weighted sum of the target cell states:

$$E[F^t(\mathbf{x}_i)] = \sum_j T_{ij}\mathbf{x}'_j$$

where $\mathbf{x}'_j$ denotes the state of target cell j at time t_1. This formulation allows us to continuously map the expected developmental flow of any initial state using the inferred global transition probabilities.

(5) Function Sensitivity Estimation

Jacobian estimation of flow map

A central objective is to understand how perturbations at an initial time influence biologically meaningful outcomes at a later time. This question is formalized through the sensitivity of the developmental flow map. For a flow map F^t mapping an initial state $\mathbf{x}_0 \in \mathbb{R}^g$ to a future state at time t, the sensitivity is captured by its Jacobian matrix $J_{F^t}(\mathbf{x}_0) = \nabla F^t(\mathbf{x}_0) \in \mathbb{R}^{g\times g}$.

To achieve a stable approximation from sparse data, we estimate the Jacobian matrix $\mathbf{A} \approx J_{F^t}(\mathbf{x}_i)$ by mapping the finite differences between pairs of cells (j,k) within the local neighborhood $\mathcal{N}_i$ (Supp. Figure-S1). Let $\Delta\mathbf{x}_{jk} = \mathbf{x}_j - \mathbf{x}_k$ denote the difference in their initial observed states, and $\Delta\mathbf{y}_{jk} = F^t(\mathbf{x}_j) - F^t(\mathbf{x}_k)$ denote the difference in their expected future states as computed by the flow map. We infer the regularized local Jacobian, denoted as $\widehat{J}_{F^t}(\mathbf{x}_i)$, by solving the following ridge regression problem:

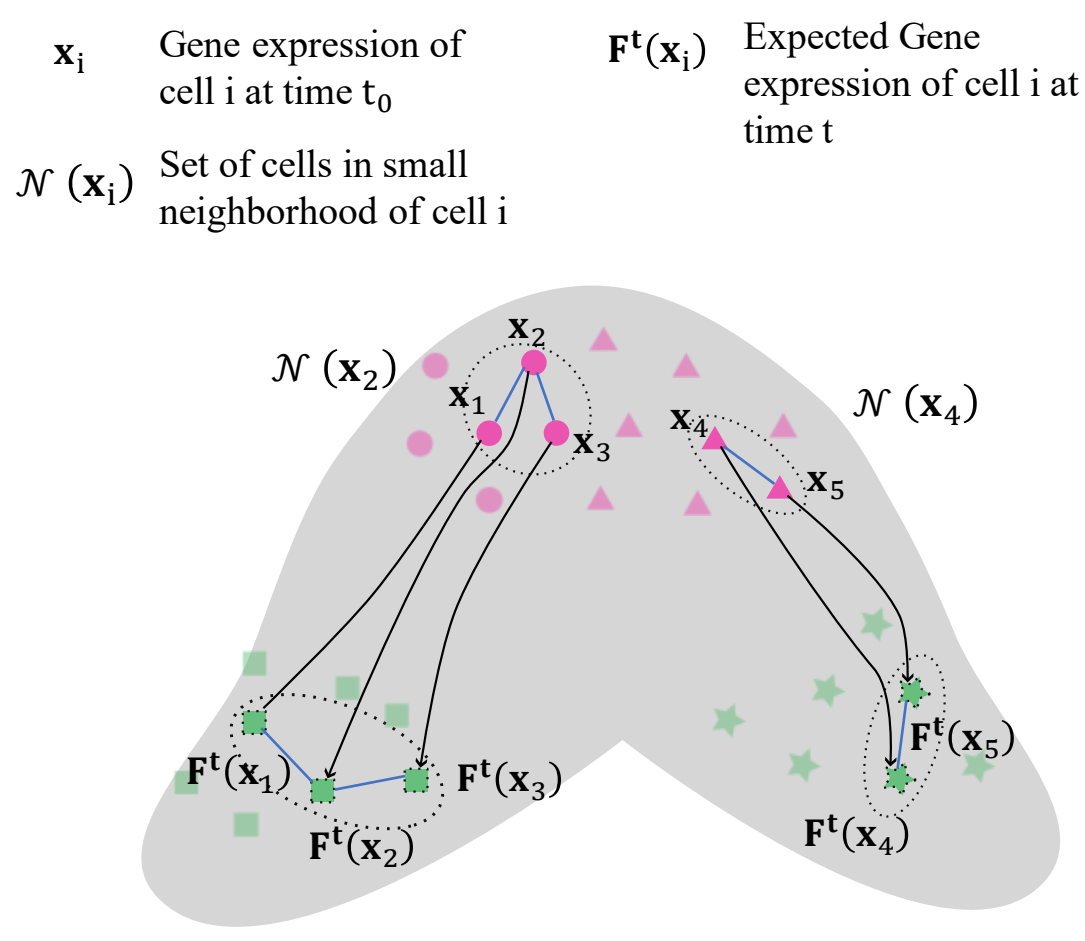

Figure S1 Schematic of Jacobian estimation. Illustrates how a cell ($\mathbf{x}_i$) and its local neighborhood map from an initial time t_0 to their expected future states ($F^t(\mathbf{x}_i)$) at time t.

$$\widehat{J}_{F^t}(\mathbf{x}_i) = \arg\min_{\mathbf{A}} \left(\sum_{j\neq k\in\mathcal{N}_i} \|\mathbf{A}\Delta\mathbf{x}_{jk} - \Delta\mathbf{y}_{jk}\|_2^2 + \beta\|\mathbf{A}\|_F^2 \right)$$

where $\beta > 0$ is a Tikhonov regularization parameter that penalizes the Frobenius norm of $\mathbf{A}$.

Functional sensitivity estimation from Jacobian of flow map

While the Jacobian of the flow map describes the sensitivity of the entire transcriptomic state, our analysis targets a specific downstream feature f, such as lineage fate probability or terminal marker gene expression. To evaluate the effect of initial state variations on this targeted quantity, we examine the composition of the function f with the flow map F^t.

Let $\mathbf{X} = F^t(\mathbf{x}_0)$ denote the expected terminal state. The local sensitivity $S_f(\mathbf{x}_0)$ is computed by multiplying the gradient of the downstream function f, evaluated at the expected terminal state $\mathbf{X}$, by the Jacobian of the flow map F^t, evaluated at the initial state $\mathbf{x}_0$:

$$S_f(\mathbf{x}_0) = \frac{\partial f}{\partial \mathbf{x}_0} = \frac{\partial f}{\partial \mathbf{X}}\frac{\partial F^t}{\partial \mathbf{x}_0} = (\nabla_{\mathbf{X}} f(\mathbf{X}))^T J_{F^t}(\mathbf{x}_0)$$

Through this matrix multiplication, we effectively project the full, high-dimensional sensitivity of the developmental trajectory onto the specific biological dimensions of interest defined by f.

To measure the commitment to a single lineage (e.g., fate 1), we define the downstream function as the norm of the terminal state projected onto a specific set of lineage marker genes: $f_1(\mathbf{X}) = \|C_{\text{fate1}}\mathbf{X}\|_2$. Here, C_{fate1} represents a projection matrix that isolates the features relevant to fate 1. The gradient of this scalar function with respect to the terminal state $\mathbf{X}$ is calculated as:

$$\nabla_{\mathbf{X}} f_1(\mathbf{X}) = \frac{C_{\text{fate1}}^T C_{\text{fate1}}\mathbf{X}}{\|C_{\text{fate1}}\mathbf{X}\|_2}$$

When evaluating the bifurcation between two distinct lineages—such as the neutrophil-monocyte split—we are interested in the relative

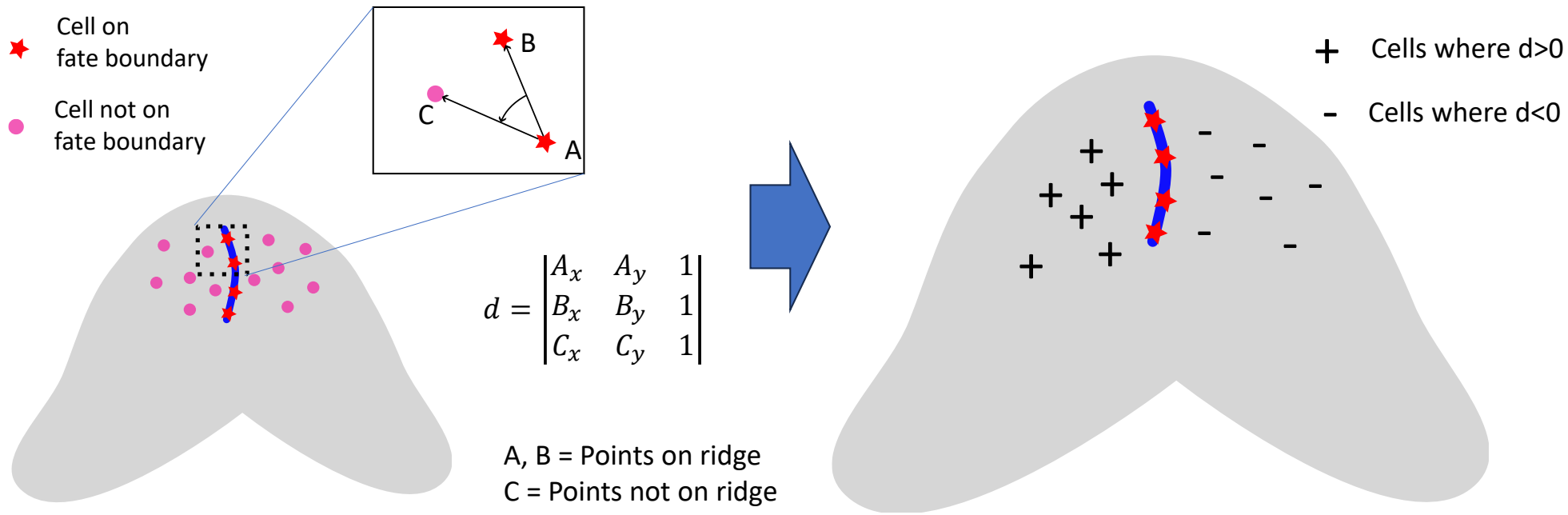

Figure S2 Schematic representation of the orientation test utilized for spatial clustering relative to a fate boundary. Points A and B are defined as reference coordinates located on the fate boundary. Point C represents a target cell that is not located on the boundary. The spatial orientation of point C is evaluated by calculating the determinant, d, of the coordinate matrix. The resulting sign of d partitions the cellular space, enabling the classification of cells into distinct regions where $d > 0$ (+) and $d < 0$ (-).

difference in commitment. We define this fate bias as $f_{\text{bias}}(\mathbf{X}) = \|C_{\text{fate1}}\mathbf{X}\|_2 - \|C_{\text{fate2}}\mathbf{X}\|_2$. By the linearity of differentiation, the gradient of this composite function is simply the difference of the individual lineage gradients:

$$\nabla_{\mathbf{X}} f_{\text{bias}}(\mathbf{X}) = \frac{C_{\text{fate1}}^T C_{\text{fate1}}\mathbf{X}}{\|C_{\text{fate1}}\mathbf{X}\|_2} - \frac{C_{\text{fate2}}^T C_{\text{fate2}}\mathbf{X}}{\|C_{\text{fate2}}\mathbf{X}\|_2}$$

By substituting this gradient into our previously established chain rule framework, we obtain the exact local sensitivity of the fate bias with respect to the initial cellular state $\mathbf{x}_0$. Treating the combined gradient as a row vector, the sensitivity $S_{f_{\text{bias}}}(\mathbf{x}_0)$ becomes:

$$S_{f_{\text{bias}}}(\mathbf{x}_0) = \left(\frac{C_{\text{fate1}}^T C_{\text{fate1}}\mathbf{X}}{\|C_{\text{fate1}}\mathbf{X}\|_2} - \frac{C_{\text{fate2}}^T C_{\text{fate2}}\mathbf{X}}{\|C_{\text{fate2}}\mathbf{X}\|_2} \right)^T J_{F^t}(\mathbf{x}_0)$$

This final matrix multiplication maps how infinitesimally small transcriptomic changes at the progenitor stage $\mathbf{x}_0$ strictly dictate the ultimate push toward either fate 1 or fate 2.

(6) Gene set enrichment analysis of regulatory genes

To biologically characterize the four regulatory gene classes identified by our sensitivity analysis, we performed Gene Set Enrichment Analysis (GSEA). The identified driver genes were partitioned into four distinct sets based on the signs of their sensitivity scores for the monocyte and neutrophil lineages: shared promoters $(+,+)$, neutrophil-specific promoters $(-,+)$, shared repressors $(-,-)$, and monocyte-specific promoters $(+,-)$. Enrichment analysis was conducted using the Enrichr(Fang et al., 2023) tool, implemented via the `gseapy` Python package. Each of the four regulatory gene lists was queried against the *MSigDB Hallmark 2020*(Liberzon et al., 2015) reference gene sets for the *Mus musculus* genome. We extracted the top five most significantly enriched functional pathways for each regulatory group, ranked by their FDR (false discovery rate) adjusted p-values.

(7) Inferring partitioning induced by Fate Boundary using an orientation test algorithm

To partition cellular data relative to the fate boundary, we implement a orientation test. As illustrated in Figure-S2, if we have points A and B as reference coordinates situated directly on the boundary and point C as a target cell located off the fate boundary. To determine the orientation of C relative to the boundary, we compute the determinant, d, of a 3×3 matrix containing the two-dimensional coordinates of these three points:

$$d = \begin{vmatrix} A_x & A_y & 1 \\ B_x & B_y & 1 \\ C_x & C_y & 1 \end{vmatrix} \tag{5}$$

The sign of this determinant dictates the grouping of the dataset. Specifically, the domain is partitioned into positive regions, denoting cells where $d > 0$, and negative regions, representing cells where $d < 0$.

Algorithm 1 formalizes this geometric principle to scale across complex spatial datasets. For a given set of boundary coordinates, R, and unassigned cells, S, the algorithm dynamically identifies the local boundary direction vector by finding the two boundary points nearest to each respective target cell. The relative spatial position is then evaluated via a cross-product, which is mathematically analogous to the determinant calculation described above. Finally, the cells are assigned to distinct clusters based strictly on the polarity of the resulting signed distance.

(8) Fate Probability Sensitivity Estimation as a constrained optimization problem

To characterize the local dynamics of lineage commitment, we examine the sensitivity of fate probabilities to gene expression perturbations. We define the fate probability sensitivity for a cell starting in state $\mathbf{x}_i$ as $A = \nabla P(\mathbf{x}_i) \in \mathbb{R}^{k \times G}$, where k is the number of distinct cell fates.

The conservation of total probability imposes a hard constraint on the Jacobian. Differentiating the simplex normalization condition $\sum_{\ell=1}^{k} P_\ell(\mathbf{x}) = 1$ with respect to the gene expression vector $\mathbf{x}$ yields:

$$\mathbf{1}^\top A = \mathbf{0}^\top \tag{6}$$

where $\mathbf{1}$ denotes the k-dimensional vector of ones.

Standard finite-difference methods for estimating A are ill-posed due to the sparsity and noise inherent in scRNA-seq data. Consequently, we formulate a regularized least-squares optimization problem within a local neighborhood $\mathcal{N}_i$. Let D_X represent the

Algorithm 1 Clustering using Sorted Orientation Test

Require: Set of boundary coordinates $R \subset \mathbb{R}^n$, Set of query coordinates $S \subset \mathbb{R}^n$, Look-ahead window $k \in \mathbb{Z}^+$
Ensure: Cluster assignments $C(s)$ for all $s \in S$
Find the starting boundary point farthest from the origin:
  $r_{start} \leftarrow \arg\max_{r \in R} \|r\|_2$
Sort boundary points R via DFS on spatial graph starting from r_{start}:
  $R_{sorted} \leftarrow \{r_1, r_2, \dots, r_n\}$, where $r_1 = r_{start}$ and $n = |R|$
for all $s \in S$ **do**
  Find the index i of the nearest boundary point $B_1 \in R_{sorted}$:
    $i \leftarrow \arg\min_{j \in \{1,\dots,n\}} \|s - r_j\|_2$
    $B_1 \leftarrow r_i$
  Define look-ahead indices to compute macro-direction:
    $i_{start} \leftarrow \min(i, n-k)$
    $i_{end} \leftarrow \min(i+k, n)$
  Compute the local boundary direction vector V:
    $V \leftarrow r_{i_{end}} - r_{i_{start}}$
  Compute vector from B_1 to spot s:
    $U \leftarrow s - B_1$
  Calculate signed distance d via cross-product:
    $d \leftarrow U_x V_y - U_y V_x$
  Cluster based on the sign of d:
  if $d > 0$ **then**
    $C(s) \leftarrow$ "Cluster 1"
  else
    $C(s) \leftarrow$ "Cluster 2"
  end if
end for
return C

matrix of gene expression displacements and D_P the corresponding fate probability differences between cell i and its nearest neighbors. We seek a linear operator A that minimizes the reconstruction error of the local mapping while strictly adhering to the probability simplex constraint.

The objective function, incorporating Tikhonov regularization, is given by:

$$\min_A \left(\frac{1}{2}\|AD_X - D_P\|_F^2 + \frac{\beta}{2}\|A\|_F^2 \right) \quad \text{subject to} \quad \mathbf{1}^\top A = \mathbf{0}^\top \tag{7}$$

where β serves as the regularization hyperparameter (the $\frac{1}{2}$ factors are introduced for analytical convenience during differentiation).

To enforce the linear constraint $\mathbf{1}^\top A = \mathbf{0}^\top$, we introduce a vector of Lagrange multipliers $\lambda \in \mathbb{R}^G$. The Lagrangian is formulated as:

$$\mathcal{L}(A, \lambda) = \frac{1}{2}\|AD_X - D_P\|_F^2 + \frac{\beta}{2}\|A\|_F^2 + \mathbf{1}^\top A\lambda \tag{8}$$

The optimality condition with respect to A is obtained by setting the gradient $\frac{\partial \mathcal{L}}{\partial A} = 0$, which yields:

$$(AD_X - D_P)D_X^\top + \beta A + \mathbf{1}\lambda^\top = 0 \tag{9}$$

Rearranging this expression to isolate the terms involving A gives:

$$A(D_X D_X^\top + \beta I) + \mathbf{1}\lambda^\top = D_P D_X^\top \tag{10}$$

By taking the transpose of this optimality condition and pairing it with the transposed simplex constraint $A^\top \mathbf{1} = \mathbf{0}$, we obtain a complete system of linear equations for the constrained estimator. This system can be compactly written in block matrix form as:

$$\begin{bmatrix} D_X D_X^\top + \beta I & \mathbf{1} \\ \mathbf{1}^\top & 0 \end{bmatrix} \begin{bmatrix} A^\top \\ \lambda^\top \end{bmatrix} = \begin{bmatrix} D_X D_P^\top \\ 0 \end{bmatrix} \tag{11}$$

Solving this linear system directly provides the constrained Jacobian estimator $\widehat{\nabla P}(x) = A$, ensuring that first-order perturbations mathematically preserve the total probability mass across the competing cell fates.

References

Z. Fang, X. Liu, and G. Peltz. Gseapy: a comprehensive package for performing gene set enrichment analysis in python. *Bioinformatics*, 39(1):btac757, 2023.

A. Liberzon et al. The molecular signatures database hallmark gene set collection. *Cell systems*, 1(6):417–425, 2015.

G. Schiebinger et al. Optimal-transport analysis of single-cell gene expression identifies developmental trajectories in reprogramming. *Cell*, 176(4):928–943, 2019.

C. Weinreb et al. Lineage tracing on transcriptional landscapes links state to fate during differentiation. *Science*, 367(6479): eaaw3381, 2020.

Y. Zhang et al. sconce: a cosmic web finder for spherical and conic geometries. *Monthly Notices of the Royal Astronomical Society*, 517(1):1197–1217, 2022.
